# How the sunflower gets its rings

**DOI:** 10.7554/eLife.86284

**Published:** 2023-02-28

**Authors:** Young-Joon Park, Pil Joon Seo

**Affiliations:** 1 Department of Chemistry, Seoul National University Seoul Republic of Korea; 2 Plant Genomics and Breeding Institute, Seoul National University Seoul Republic of Korea

**Keywords:** *Helianthus annuus*, floral maturation, anthesis, spiral phyllotaxy, rhythms, floral organs, Other

## Abstract

The circadian clock may help to control the development patterns which allow the florets on a sunflower head to go through their final stages of maturation at precisely the right time.

**Related research article** Marshall CM, Thompson VL, Creux NM, Harmer SL. 2023. The circadian clock controls temporal and spatial patterns of floral development in sunflower. *eLife* **12**:e80984. doi: 10.7554/eLife.80984.

Development requires intricate processes to occur at the right time and in the right place so that organs can take shape and mature properly. These pathways are under the control of well-designed genetic programs which can coordinate spatial and temporal information across the organism. Exactly how this integration takes place remains an important biological question.

The sunflower is a good model in which to study how floral development is spatially and temporally regulated, as its flowering head is comprised of thousands of individual florets that display a unique pattern of maturation. During early development, these tiny flowers are organised in a continuous spiral, with the youngest at the centre and the oldest at the periphery (Marc and Palmer, 1981). In later developmental stages, the florets enter a period known as anthesis: every day for the next five to ten days they become sexually mature in order to release and receive pollen.

This process starts in the outer region, with hundreds of florets simultaneously undergoing anthesis in a discrete ring-like structure known as a pseudowhorl. Every day, a new ‘circle’ of florets matures and another pseudowhorl forms (Baroncelli et al., 1990; Lobello et al., 2000). However, the mechanisms which allow an early continuous ‘spiral’ pattern to be converted into discrete pseudowhorls during floral maturation have so far remained elusive. Now, in eLife, Stacey Harmer and colleagues at the University of California, Davis and the University of Pretoria – including Carine Marshall as first author – report that this change in pattern may be under the control of circadian rhythms (Marshall et al., 2023).

The team first aimed to examine the daily rhythms of floral development. To do so, they grew sunflowers at stable temperatures under normal daylight patterns, and dissected florets from a single ring every 15–30 minutes to examine their organs. This manipulation showed that, within a floret, male and female sexual structures develop following a precise 24 hour pattern which prevents the organs from being fully mature at the same time. The experiment also showed that florets of different ages all matured in a remarkably coordinated fashion across the pseudowhorl, which resulted in the production of the discrete ring-like structures on the sunflower head (Figure 1A).

**Figure 1.**
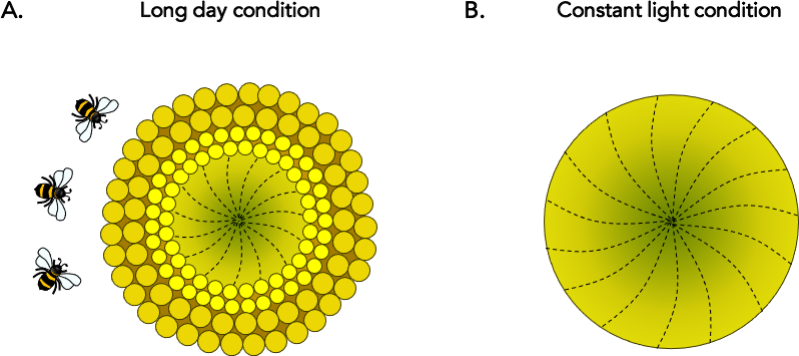
Circadian control of floral development in sunflowers. (**A**) During the early stages of development, the individual florets of the sunflower develop in a continuous age-dependent manner to form a spiral (dotted lines) across the ‘head’ of the plant: this results in the youngest florets being located at the centre (dark green) and the older florets at the periphery (light green). Normal daylight patterns, including those featuring extended periods of light (‘long day’), preserve circadian robustness. In these cases, a series of concentric ring-like developmental zones, or ‘pseudowhorls’, are established every 24 hours; the first (large dark yellow circles) emerges at the outer edge of the plant, followed by another more internal ring the next day (small bright yellow circles), and so on. Maturation of individual florets is highly synchronized within a pseudowhorl, which explains how their organization transitions from a continuous spiral to discrete rings. The unique patterns of anthesis may optimize plant-pollinator interactions, ensuring plant reproductive success. (**B**) Without robust circadian oscillation (that is, under constant light conditions), the characteristic floral development patterns disappear and no pseudowhorls are generated. These plants attract fewer pollinators in the field.

For Marshall et al., these intricate daily rhythms indicate that the circadian clock may be involved in controlling the timing of anthesis in sunflowers. This genetic timing mechanism allows biological rhythms to take place every 24 hours and at the right time. While this clock uses light and other cues to ensure that an organism remains synchronized with the night and day cycle, it is also capable of maintaining biological rhythms for some time even when certain daily cues are disrupted (Sabrina and Steve, 2016).

To test their hypothesis, Marshall et al. recorded sunflowers maturing under various light and temperature conditions. The experiments showed that floral maturation remained unaffected as long as the environmental changes did not impact circadian rhythms. For example, sunflowers exposed to various temperatures or even constant darkness retained strong circadian cycles: these plants still formed new pseudowhorls day after day, and floret growth within each ring remained synchronized (Figure 1A). This result supports the idea that the circadian clock is involved in coordinating floral development (Peter et al., 2006).

However, the characteristic anthesis patterns disappeared when sunflowers were grown under constant light, which was a condition that disrupted circadian rhythms (Figure 1B). In these plants, late developmental stages tended to resemble earlier ones: the florets did not form discrete pseudowhorls but instead aged in a continuous spiral. Together, these results demonstrate that, in sunflowers, circadian coordination is required for pseudowhorls to form at the late floral developmental stage. The genetic pathways which determine when and how these plants flower may therefore be regulated by the molecular mechanisms of the circadian clock.

Next, Marshall et al. suggested that circadian control of floret development likely promotes reproductive success in sunflowers. To test this theory, they conducted a series of experiments in more relevant ecological conditions. When sunflowers grown under constant light which lacked pseudowhorls were placed in a field at dawn, they were visited by fewer pollinating insects than their unaltered counterparts (Figure 1A). This was also the case for plants in which anthesis had been artificially manipulated to occur later in the day. According to previous reports, bees are more attracted to sunflowers which have higher numbers of maturing florets (DeGrandi-Hoffman and Watkins, 2000; Neff and Simpson, 1990); individual florets all becoming sexually ready at the same time on successive days may therefore help pollinators to predict floret development and coordinate their visit to the plant. In addition, male and female organs maturing at different times of the day within a single flower is an excellent strategy to avoid self-pollination. Anthesis and pseudowhorl formation being dependent on the circadian clock may therefore enhance reproductive success in sunflower.

Molecular evidence is now needed to further support the conclusions drawn by Marshall et al. For instance, it is currently unclear how circadian and environmental signals are integrated; exactly which molecular actors from the circadian clock participate in the spatiotemporal coordination of floral development also remains unresolved. Finally, additional work will be required to dissect the mechanisms that allow florets over a certain age to mature every 24 hours. Answering these questions will help to better understand how floral development relies on circadian rhythms. In turn, this knowledge could be useful for creating new varieties of crops which are optimised for pollination in fluctuating natural environments.

## References

[bib1] Baroncelli S, Lercari B, Cecconi F, Pugliesi C (1990). Light control of elongation of filament in sunflower (*Helianthus annuus L.*). Photochemistry and Photobiology.

[bib2] DeGrandi-Hoffman G, Watkins JC (2000). The foraging activity of honey bees *Apis mellifera* and non-apis bees on hybrid sunflowers (*Helianthus annuus*) and its influence on cross-pollination and seed set. Journal of Apicultural Research.

[bib3] Lobello G, Fambrini M, Baraldi R, Lercari B, Pugliesi C (2000). Hormonal influence on photocontrol of the protandry in the genus *Helianthus*. Journal of Experimental Botany.

[bib4] Marc J, Palmer JH (1981). Photoperiodic sensitivity of inflorescence initiation and development in sunflower. Field Crops Research.

[bib5] Marshall CM, Thompson VL, Creux NM, Harmer SL (2023). The circadian clock controls temporal and spatial patterns of floral development in sunflower. eLife.

[bib6] Neff JL, Simpson BB (1990). The roles of phenology and reward structure in the pollination biology of wild sunflower (*Helianthus annuus L.*, Asteraceae). Israel Journal of Botany.

[bib7] Peter DG, James CW, Camille L, Megan MS, Seth JD, Shigeru H, Richard M, Raechel M, Joanna P, Andrew JM, Anthony H (2006). The molecular basis of temperature compensation in the *Arabidopsis* circadian clock. The Plant Cell.

[bib8] Sabrina ES, Steve AK (2016). The plant circadian clock: from a simple timekeeper to a complex developmental manager. Cold Spring Harbor Perspectives in Biology.

